# Kairomone-like activity of bile and bile components: A step towards revealing the chemical nature of fish kairomone

**DOI:** 10.1038/s41598-020-63456-z

**Published:** 2020-04-27

**Authors:** Joanna Pijanowska, Magdalena Markowska, Anna Ruszczyńska, Ewa Bulska, Piotr Dawidowicz, Mirosław Ślusarczyk, Magdalena Biesaga

**Affiliations:** 1 Department of Hydrobiology, Faculty of Biology, Biological and Chemical Research Centre, University of Warsaw, Żwirki i Wigury 101, 02-089, Warsaw, Poland; 2 Department of Animal Physiology, Faculty of Biology, University of Warsaw, Miecznikowa 1, 02-096, Warsaw, Poland; 3 Faculty of Chemistry, Biological and Chemical Research Centre, University of Warsaw, Żwirki i Wigury 101, 02-089, Warsaw, Poland; 4 Faculty of Chemistry, University of Warsaw, Pasteura 1, 02-093, Warsaw, Poland

**Keywords:** Chemical biology, Ecology, Evolution, Limnology

## Abstract

Despite the efforts of a number of research groups worldwide, we still have a poor understanding of the chemical nature of the fish kairomones which induce defensive morphology, life history and behavior in their planktonic prey. Bile excreted by foraging fish play a crucial role in their signaling systems. Using high-performance liquid chromatography (HPLC), we revealed the presence of primary and secondary bile acids and bile salts in fish-conditioned water, similar as in carp bile. Upon exposure to either fish bile or commercially acquired bile salts, *Daphnia* demonstrated similar changes in life history and behavior as when exposed to fish kairomones. The synergic effect of the injured *Daphnia* alarm substance with fish bile on *Daphnia* life history is similar to the adaptive effect of the same alarm substance combined with fish kairomones. This strongly supports the view that fish bile or selected bile acids/salts may be responsible for the biological activity of kairomones.

## Introduction

In the aquatic realm, organisms are confronted with a plethora of kinds of chemical information, originating from their live food, competitors, predators and conspecifics. The ability to recognize this information can be crucial for the fitness of these organisms. Planktonic animals rely on their ability to recognize chemical traces of predator presence (so called kairomones) to identify the nature of the threat. Fish-conditioned water has the ability to directly induce defensive behavioral (e.g. diel vertical migrations), life history (e. g. age at first reproduction, offspring number, reproductive effort and diapause), and morphological changes^1–3^. Despite the concerted efforts of a number of research groups worldwide, so far the chemical nature of the fish and invertebrate kairomones which induce changes in their zooplankton prey remains unrecognized^4^. Identification of kairomone structure seems to be crucial for further research on predator-prey relationships in aquatic habitats. Resolving the frequent problem of contradictory results obtained in various experiments by different authors or determining the concentration of kairomone in the wild to assess the strength of fish pressure will only become possible with the standardized methods based on known kairomone structure and concentration. Previous studies revealed that fish kairomone is a non-volatile substance with a low molecular weight (<500 Da), and contains an anionic compound of medium polarity. Cyprinid kairomones were demonstrated to exhibit a high degree of stability within a broad range of pH (0.8 < pH < 14.0) and temperature (−20 °C < T < 120 °C) conditions and insensitiveness to proteinase, alkaline phosphatase, glucuronidase and sulphatase^5–7^. Moreover, polar hydroxyl groups have been revealed to be essential for the biological activity of kairomones^6,7^. Due to microbial degradation, a water-soluble kairomone ceases all of its activity under non-sterile conditions at 37 °C^5^, its activity being impaired by planktonic bacteria^8^. Basing on a comparison of the FTIR (Fourier-transform infrared spectroscopy) spectra of fish-conditioned and control water, Akkas and al. (2010) suggested that an interaction between an N-H, a hydroxyl and a methyl group is essential for kairomone activity. They confirmed previous reports that phosphate groups, peptidoglycan lipids and lipopolisacharydes do not contribute to this process. Instead of proteins, they proposed N-H groups from amine molecules as crucial for the biological activity of the kairomone.

Trimethylamine (TMA), which was previously reported as a key fish kairomone factor^9^, has eventually been excluded as it lacks N-H bonds, its effect on diel vertical migrations (DVM) was shown to have been overestimated^10^ and no effect on life history was detected^11^.

The molecules responsible for kairomone activity are likely to be evolutionarily conservative, and these substances should have a relatively unstable presence in the environment, being continuously degraded and replaced and, of course, indicative of predation pressure only when and where fish are present.

For more than a decade an increasing amount of research has been published on intraspecific communication between fish, utilizing a combination of bile acids and bile salts as a multipotent signal that induces a number of essential life processes in fish, such as foraging behavior, detecting nearby fish, assessing risk, mating, homing, spawning and migratory behavior^12^. In addition to their primary known function as lipid digestion agents, the important role they play in fish lives as complex signalling molecules has recently been appreciated. Together with amino acids, sex steroids and prostaglandins, bile acids/salts constitute one of the four major classes of chemicals that have been identified as specific olfactory stimuli in fish^13^. Reproductively mature male sea lampreys (*Petromyzon marinus*) release a bile acid which acts as sex pheromone, inducing long-distance searching behavior in ovulating females^14^. Adult lampreys also use a bile acid-based larval pheromone to locate spawning rivers^15^. A number of similarities exists between fish and insects in e.g. the anatomy of their olfactory pathways and in information processing circuits^16^. This indicates that the information processing pathway may be highly conservative in animals. Bile salts are released by fish into the water via the intestine, urinary tract and gills, adding to the richness of semiochemicals in aquatic habitats and possibly inducing various reactions in potential receivers^12^.

The dominant component of bile in ray-finned fish (Actinopterygii) are common C24 bile acids: cholic and chenodeoxycholic acid and, in the *Cypriniformes*, also 5αC27 bile alcohol, known as cyprinol is present^17,18^. In fish, bile acids are conjugated mostly with taurine^17^. Recently, Li *et al*. ^19^ quantified 15 bile acids and found detectable amounts of cholic acid (CA), taurochenodeoxycholic acid/salt (TCDCA), taurocholic acid/salt (TCA) and chenodeoxycholic acid/salt (CDCA) in the faeces of lake charr *(Salvelinus namaycush)*. Since bile salts are commonly used in the complex signaling systems of fish, they should presumably be present in lake water, most probably in greater concentrations during the warm seasons, when fish foraging is the most effective. Therefore, first, we investigated if bile and two of its most common components (cholic acid and deoxycholic acid) induce adaptive life history and behavioral changes similar to those induced by the fish kairomone in potential fish prey - cladoceran *Daphnia magna* and, second, we checked for the presence of primary and secondary bile acids and bile salts (known as ‘components of bile’, listed in Table 1) in fish-conditioned water, using high-performance liquid chromatography (HPLC). Our focus on bile did not stem from a blind search; we were concentrating our efforts, the results of which were first demonstrated by Markowska *et al*. ^20^ at the XI Symposium on Cladocera in Kulmbach on those candidate compounds which are released into water by fish and have become increasingly important in their evolution, being involved in both intra- and interspecific communication. Following our preliminary results, Hahn *et al*. ^21^ confirmed kairomone-like activity of some components of fish bile.

**Table 1:** Bile acids, salts and alcohol analyzed in presented paper.

bile compound	primary/secondary acid	abbreviation
cholic acid	primary	CA
glycocholic acid	primary	GCA
taurocholic acid	primary	TCA
chenodeoxycholic acid	primary	CDCA
ursodeoxycholic acid	secondary	UDCA
hyodeoxycholic acid	secondary	HDCA
deoxycholic acid	secondary	DCA
taurodeoxcholic acid	secondary	TDCA
taurohyodeoxycholic acid	secondary	THDCA
tauroursodeoxycholic acid	secondary	TUDCA
glycodeoxycholic acid	secondary	GDCA
glycochenodeoxycholic acid	primary	GCDCA
5α-cyprinol	alcohol	cyprinol

The liver synthesizes cholate and chenodeoxycholate (primary bile acids) from cholesterol. The intestinal bacteria convert some of them into secondary bile acids such as deoxycholate and lithocholate. Conjugation with taurine or glycine occurs in the liver and bile salts are secreted into the bile in this form. According to Hoffman *et al*. (2010)^17^, we will be using the term „bile salts” for bile acids conjugated with taurine or glycine as well as for sodium salts.

## Results

### Bile and bile salts induce changes in the life history parameters of *Daphnia magna*

**Figure 1:**
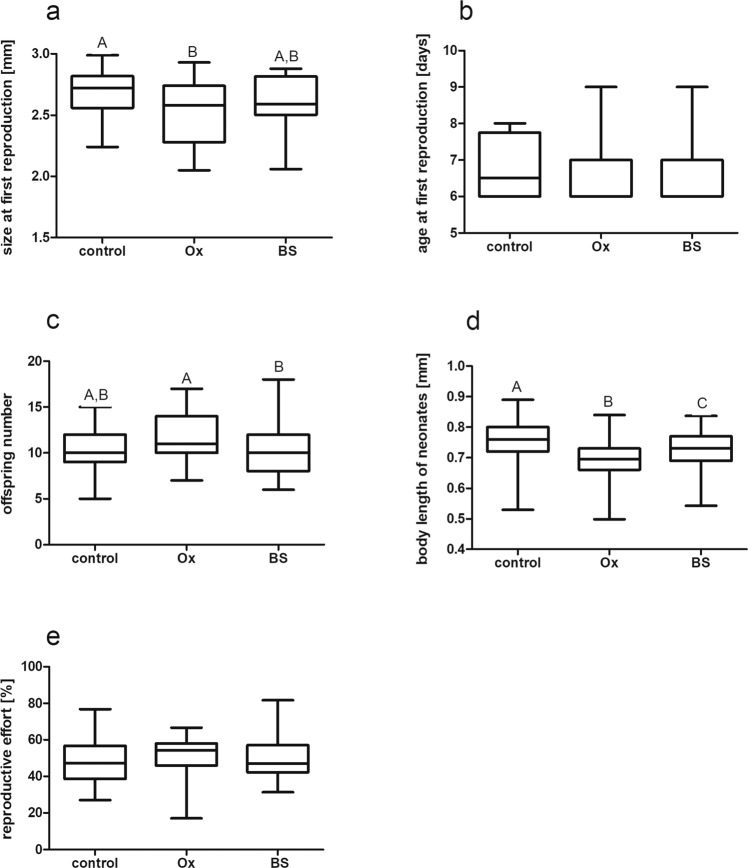
Bile and bile salts induce changes in the *Daphnia magna* life history. Effect of ox bile (Ox) and bile salts (BS) on *D. magna* life history: size at first reproduction (**a**), age at first reproduction (**b**), offspring number (**c**), body length of neonates (**d**) and reproductive effort (**e**). As a reference, we show control treatment with no addition of any compounds (control). Results are presented as boxes bisected at the median value with minimal and maximal whiskers. Letters indicate statistically significant differences between groups. Results of Kruskall-Wallis (H and *p*) as well as *p* of Dunn’s post hoc test are presented in the Table S2 in the Supporting information.

**Figure 2:**
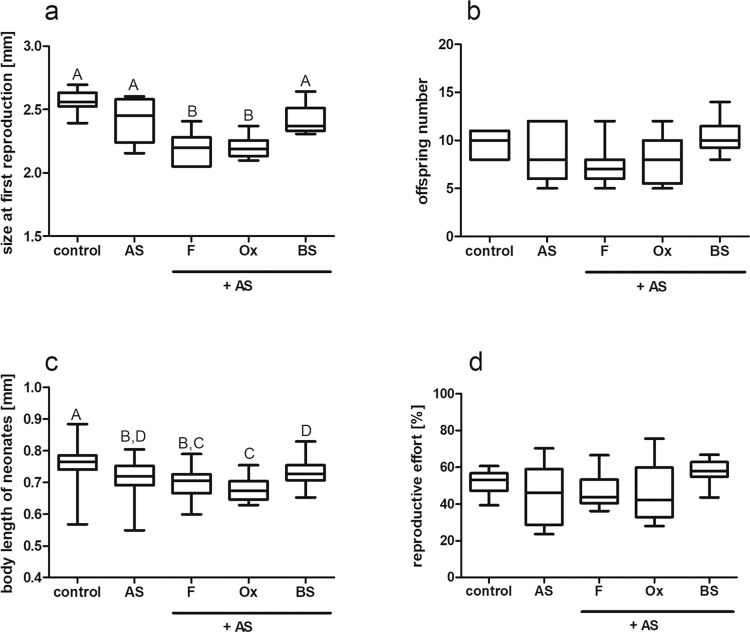
Fish kairomone, bile and bile salts induce changes in the *Daphnia magna* life history, modified by alarm substance. Effect of fish kairomone (F), ox bile (Ox) and bile salts (BS) with the addition of alarm substance (AS) on *D. magna* life history: size at first reproduction (**a**), offspring number (**b**), body length of neonates (**c**) and reproductive effort (**d**). As a reference, we show control treatment with no addition of any compounds (control) and sole alarm substance. Results are presented as boxes bisected at the median value with minimal and maximal whiskers. Letters indicate statistically significant differences between groups. Results of Kruskall-Wallis (H and p) as well as p of Dunn’s post hoc test are presented in Table S3 in the Supporting information.

We found significant differences between the control, ox bile (Ox) and bile salts (BS) treatments in 2 out of the 5 measured *Daphnia* life history parameters (Fig. 1). In the presence of ox bile, but not bile salts, body size at first reproduction was significantly smaller than in the control treatment (Fig. 1a). The insufficient time resolution (every 24 hours) of measuring age at first reproduction did not allow us to detect potential differences between treatments for this value (Fig. 1b). Number of offspring was significantly higher in ox bile than in bile salts treatments, but neither of these treatments differed from the control (Fig. 1c). The body length of neonates differed significantly between treatments and was lower in each of the two bile treatments than in the control (Fig. 1d). The addition of ox bile (Ox) to a medium containing the chemical traces of injured *Daphnia* strengthened the effect of the pure alarm substance (AS) producing the same synergistic effect as the mixture of fish kairomone (F) and alarm substance (Fig. 2). Body size at first reproduction was significantly smaller in the presence of fish kairomone and ox bile than in the control treatment but did not differ from the control in the sole presence of alarm substance or its combination with bile salts (Fig. 2a). While no significant differences in offspring number (Fig. 2b) reproductive effort (Figs. 1e, 2d) were observed between treatments, the body length of neonates was significantly larger in the control than in all other treatments (Figs. 1d, 2c).

### Bile, bile salts and *Cyprinidae* bile have the same effects as a fish kairomone on the vertical distribution of *Daphnia magna*

**Figure 3:**
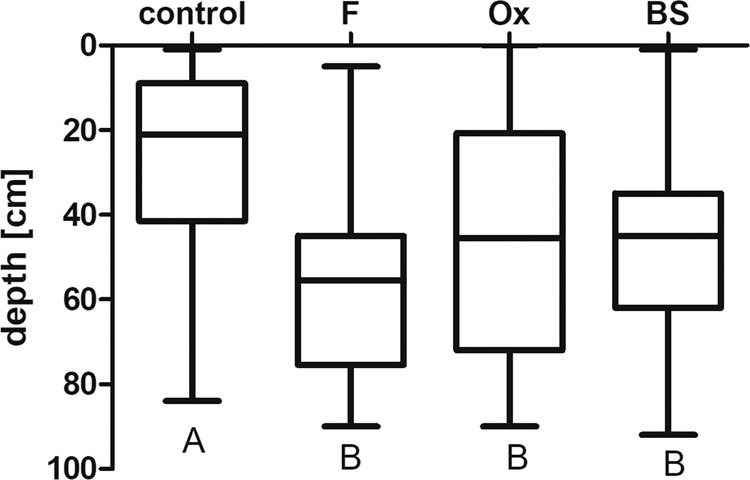
Bile and bile salts act as fish kairomone on *Daphnia magna* vertical distribution. Box plot of the depth distribution of *D. magna* exposed to fish kairomone (F), ox bile (Ox) and bile salts (BS) in the “plankton organ”. As a reference, we show control treatment with no addition of any compounds (control). Results are presented as boxes bisected at the median value with minimal and maximal whiskers. Letters indicate statistically significant differences between groups. Results of Kruskall-Wallis (H and p) as well as p of Dunn’s post hoc test are presented in Table S4 in the Supporting information.

**Figure 4:**
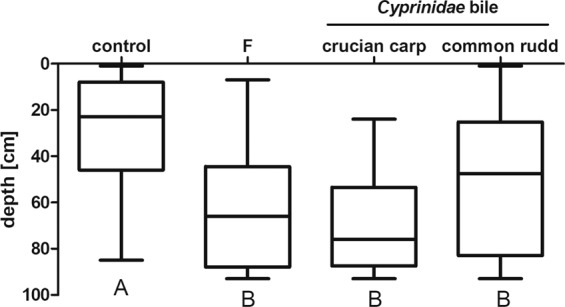
Fish bile act as fish kairomone on *Daphnia magna* vertical distribution. Box plot of the depth distribution of *D. magna* exposed to fish kairomone (F) and fish bile in the “plankton organ”. As a reference, we show control treatment with no addition of any compounds (control). Results are presented as boxes bisected at the median value with minimal and maximal whiskers. Letters indicate statistically significant differences between groups. Results of Kruskall-Wallis (H and p) as well as p of Dunn’s post hoc test are presented in Table S5 in the Supporting information.

Diurnal depth differed significantly between the treatments (Figs. 3, 4). Exposed to the presence of either fish kairomone (F) or ox bile (Ox) and bile salts (BS), *Daphnia* remained significantly deeper in the water column during the day than did the control specimens. Though each of the three experimental treatments differed significantly from the control, there were no differences between the fish, ox bile and bile salts treatments. Similarly, exposed to the presence of fish kairomone or bile freshly extracted from fish gallbladder, *Daphnia* remained significantly deeper in the water column during the day than control individuals (Fig. 4), with no differences observed between fish kairomone and fish bile regardless of the species used.

### In fish kairomone, similar bile salts and acids are present as in fish bile and ox bile

**Figure 5:**
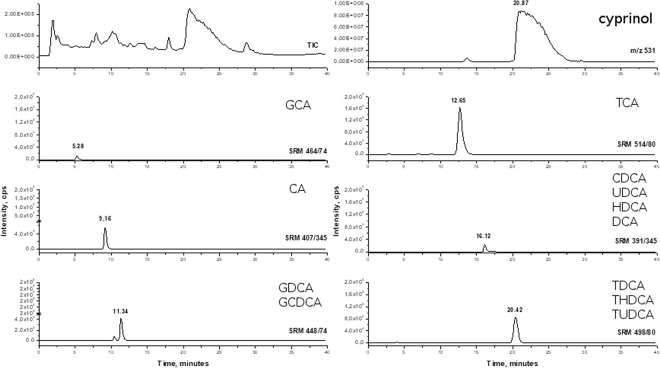
Total ion chromatogram (TIC), single ion monitoring (SIM) at m/z 531 for ion cyprinol and extracted ion chromatograms in fish conditioned water.

**Figure 6:**
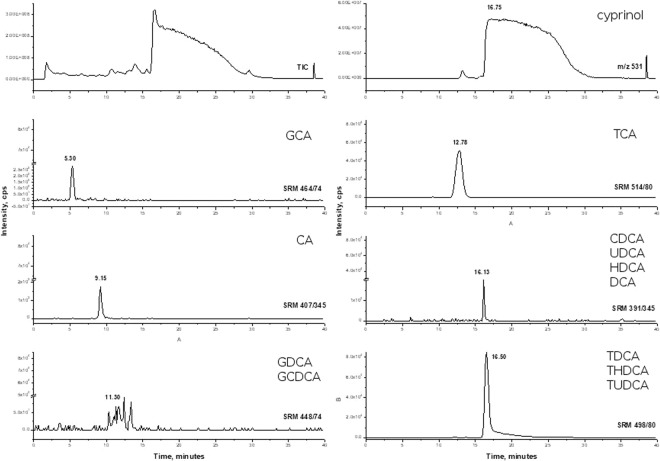
Total ion chromatogram (TIC), single ion monitoring (SIM) at m/z 531 for ion cyprinol and extracted ion chromatograms (SRM) of tested compounds in carp bile diluted 30 times (initial sample concentration 0.36 g/g).

**Figure 7:**
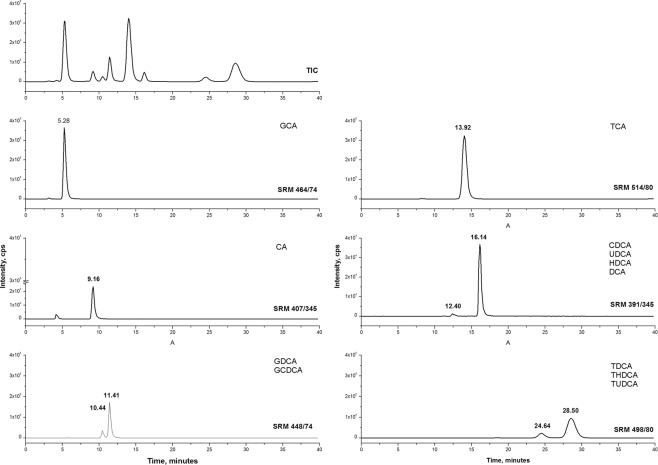
Chromatograms of total ion current (TIC) and extracted ion chromatograms (SRM) for bile acids and salts from ox bile. Sample concentration 66 mg/L.

Chromatographic analysis of the fish-conditioned water revealed the presence of all of the expected bile acids and salts (Fig. 5). Moreover, analysis revealed that the bile acids found in the fish-conditioned water correspond with those observed in the ox bile and carp bile (Figs. 5, 6 Table 2 and Supplementary T1) used in our experiments. In all of the solutions analyzed, taurine deoxycholic salts (TDCA, THDCA, TUDCA) had the same mass (i.e. the same SRM) but slightly different retention times, which may indicate the presence of different isomers of the compounds under study. As predicted, cyprinol, the bile alcohol specific for *Cypriniformes*, was present in the fish-conditioned water (Fig. 5) and the carp bile (Fig. 6), but was not detected in the ox bile (Fig. 7). The ox bile TCA also had a slightly different retention time than the TCA present in the fish-conditioned water and the carp bile.

**Table 2:** Content of bile components in fish conditioned water, ox bile, and carp bile. Presented are retention times (in minutes) of components with the same mass.

	FISH WATER	OX BILE	CARP BILE
CA	9,16	9,16	9,15
GCA	5,28	5,28	5,30
TCA	12,65	13,92	12,78
CDCA	16,12	16,14	16,13
UDCA			
HDCA			
DCA			
TDCA	20,42	24,64 and 28,50	16,50
THDCA			
TUDCA			
GDCA	11,34	10,44 and 11,41	11,30
GCDCA			
cyprinol	20,87	nd	16,75

## Discussion

When exposed to either fish bile, mammalian bile or bile salts, *Daphnia magna* demonstrated similar changes in life history (Figs. 1, 2) and behavior (Figs. 3, 4) as when exposed to fish kairomones^22^. In the face of the potential threat of fish predation, as indicated by the presence of bile or its compounds, following appear to be adaptive responses, similar to those induced by the fish kairomone: smaller body size at first reproduction, larger number of offspring, reduced neonate size and a preference to dwell deeper in the water column. These strategies are all considered to be adaptive as they offer relative safety against visual predation^23^. Although the presence of an alarm substance originating from injured *Daphnia* is not necessary to reveal bile activity, the synergic effect of the interaction of the alarm substance with bile (Fig. 2) is similar if not identical to the adaptive effect on *Daphnia* life history of fish kairomones combined with the same alarm substance. Apparently, bile excreted into water by fish has similar properties to those of fish kairomones as a potent signal, but is not necessarily identical to fish kairomones. The presence, however, of several primary and secondary bile acids and salts in fish-conditioned water as in carp bile (as revealed by chromatography and MS analyses; Figs. 5, 6) strongly supports the view that bile or selected bile acids/salts may be responsible for the biological activity of kairomones.

Many of the attempts that have been undertaken by researchers to reveal the chemical nature of fish kairomones have demonstrated the peculiar chemical properties of these substances, which are similar to those of bile and bile acids/salts^5–7,10^: they are polar, not volatile and have low molecular weight (all are smaller than 500 Da, with the largest, cyprinol, weighing 531 Da). As acids, they lack an anionic compound. They have hydroxyl groups which are necessary for their biological activity. Bile components are not proteins, so they are resistant to peptidases. They lack bonds which would degrade when exposed to driselase, cellulase, laminarinase or xylanase. In fish, bile acids are rarely conjugated with glucuronic acid; thus, they are insensitive to glucuronidase.

The effect of the mixture of cholic and deoxycholic acids/salts was less pronounced than that exerted by the bile, whether or not the *Daphnia* alarm substance was present (Fig. 2). This suggests that besides these two, other components of bile are responsible for its full activity, similar to that one originating from fish kairomones. Our results suggest that the presence of bile acids which are conjugated, most probably with taurine, may be important for the kairomone-like activity of bile. This is in line with the results of other studies which have demonstrated that the N-H bond, which is also present in taurine, is required for the biological activity of kairomones^10^. Finally, the fact that both, ox bile lacking cyprinol (an alcohol characteristic of the *Cypriformes)* and fish-conditioned water as well as fish bile in which cyprinol is present have similar effects on the life history and depth distribution of *D. magna* means that this compound is clearly not essential for kairomone-like activity. This is in contrast to recent study of Hahn and co-workers (2019)^21^ who observed cyprinol induced DVM in *Daphnia.*

In our experiment, we used a concentration of 0.001% w/v bile and bile salts. This means that we were using 1 mg of compound per individual *Daphnia* specimen in the glass, which is most likely at a much higher concentration than that occurring in lake water. Li and co-workers (2015)^19^, however, found that lake charr excreted an average of 400 ng bile acids per milligram of faeces. Given the high density of fish in lakes, especially during the spring, as well as the relative stability of the cholesterol ring, the main compound of bile salts/acids/alcohols, the concentration of bile in the water may be high and can reach mg values. In the DVM experiment with fish bile, we used lower 0.0001% v/v concentration which was as effective as ox bile. In the future, we plan to follow the seasonal dynamics of bile salts concentrations in lakes.

In mammals, it has been observed that bile acids act via specific receptors, one of them being the farnesoid X receptor^24^. Phylogenetic reconstructions show that the vertebrate farnesoid X receptor is closely related to the ecdysone receptors of insects and crustaceans^25^. This sex hormone of invertebrates has been recently proposed as being involved in the mechanism of fish kairomone action^26^.

Although bile and bile acids exert similar effect as fish kairomone on *Daphnia* life history and behavior, one cannot exclude that the kairomone itself may be a more complex cocktail, comprised not only of bile components but also of different substances (e. g. other steroids and prostaglandins) which are released into the water as byproducts of fish metabolism and are used in their communication networks.

Current theory suggests that many signaling systems have evolved from preexisting chemical cues^27^. As long as the signal is highly beneficial for the emitter, its production and releasing will be maintained by selection forces and individuals of other species may learn to parasite this information, exploiting the signaling system for their own profit. If fish are engaged in an exchange of information, their potential prey may seek to extract the information from the signaling interchange. Complex systems of interspecific communication can evolve via predator-borne cues and signal recognition by prey. The hijacking of such information by individuals other than the primary target is widespread in the aquatic environment. Eavesdropping on such an information by a third party^28^ (cladoceran prey in our scenario) may be highly beneficial since it enables the prey to detect the presence of a predator, estimate the scale of the threat, and then adequately adjust their behavior or life cycle.

In summary, our study provides further documentation of the evolutionary dynamics of predator signaling and utilization of predator borne cue by prey organisms in a complex system of predator-prey interactions. Together with the recent paper on the nature of aquatic invertebrates (*Chaoborus*) kairomone by Weiss *et al*. ^29^, our data contribute to the advancement in our knowledge on chemical communication in aquatic environment.

## Material and Methods

### Biotests

In all biotests, we used clonal females of *Daphnia magna* originating from Binnensee, a lake in North-eastern Germany (GIS coordinates 54.3256 N, 10.6296 E) inhabited by fish^30^, and maintained in a laboratory culture for over 20 years. The water used in the experiments (originating from the Szczęśliwice reservoir in Warsaw, Poland) had been stored in a 10 m^3^ underground tank for at least one month prior to the experiments. The water was aerated and filtered through a 0.45 µm filter prior to use, with microalgae *Acutodesmus obliguus* added as food for the *Daphnia* at a concentration of 1 mg of organic C/L in the life history biotest and 2 mg C/L in the vertical distribution bioassay.

### Life history bioassay: effects of bile and bile salts on the life history of *Daphnia*

In the first experiment, we investigated the life history traits crucial for fitness of *Daphnia* by comparing their reaction to three treatments labeled control, ox bile – Ox, and bile salts – BS (described below). We purchased from the Sigma Aldrich company lyophilized ox bile (# 70168) and a mixture (#48305-F) of the most common primary cholic acid (CA) and its secondary form – sodium salts of deoxycholic acid (DCA), than called bile salts. Ox bile was used because we could not obtain enough *Cyprinidae* bile for the long term life history experiments and, moreover, it was commercially unavailable. The reagents were dissolved in distilled water to 1% stock solution and stored at −20 °C. In the bile salt (BS) and ox bile (Ox) treatments, solutions of these two substances were added to water to obtain a final concentration of 0.001%. This concentration was selected from among four potential alternatives (each applied in a gradient from 1% to 0.001% through 0.1% and 0.01%) as it caused no mortality in the *Daphnia* experimental population. In this pilot study, 10 neonate *Daphnia* were individually exposed to these concentrations of bile and bile salts until their first reproduction and the number of survivors was counted daily. Later, during the proper life history bioassays, in each treatment 10 *Daphnia* were cultured individually in 100 mL of medium in 250 mL capacity glass jars. Media were changed daily to maintain a sufficient amount of food and appropriate concentrations of bile and bile salts. The experiments were run at 20 °C and a 16 L:8D photoperiod and were replicated four times, i. e. 40 specimens were examined in each treatment.

In the second experiment, we investigated whether the addition of ox bile and bile salts to media containing *Daphnia* would reinforce the effect of an alarm substance originating from homogenized conspecifics on *Daphnia* traits, as fish kairomone has been found to do^31^. This so-called alarm substance is released from mechanically damaged *Daphnia* tissues and, when applied alone, acts as nonspecific cue, advertising danger from predators and initiating a series of different behavioral and life history responses in survivors^32^. When combined with either fish or invertebrate kairomones, it serves to advertise the type and scale of danger. In this study, the alarm substance was obtained by homogenizing 250 *Daphnia* individuals in 100 mL of lake water. In the alarm substance (AS) treatment, a *Daphnia* homogenate has been added to water in a concentration of 25 *Daphnia* L^−1^. We obtained fish kairomone (F) by keeping 20 fish (crucian carp, *Carassius carassius*) for 24 hours in 10 L of lake water. The resulting stock solution was then portioned and frozen, and this frozen solution was later added to the media in a concentration of 0.1 fish L^−1^. In the bile salt (BS + AS) and ox bile (Ox + AS) treatments, the *Daphnia* alarm substance was supplemented with bile and bile salts at a concentration of 0.001%. Under each treatment, 10 *Daphnia* were kept individually in 100 mL of medium in 250 mL capacity glass jars. Media were changed daily to maintain a sufficient amount of food and appropriate concentrations of either kairomones, alarm substances or ox bile and bile salts. The experiment was run in the incubators at 20 °C and a 16 L:8D photoperiod.

In both life history bioassays, we measured the size and age at first reproduction (the latter within 24 h accuracy), counted the number of first clutch neonates and measured their length using the NIS elements program from Nikon. Reproductive effort (ratio of neonate biomass to female biomass × 100%) was calculated for all females. Weights of individual *Daphnia* were calculated from the length (L)-weight (W) relationship obtained for *D. magna* cultured in our laboratory under the same conditions (W = 2.583 × length^2.406^).

### Vertical distribution bioassay: effect of bile and bile salts on the diurnal depth distribution of *Daphnia*

The test animals were 5 day-old clonal *Daphnia magna*. To test the putative effects of kairomones on depth selection by *Daphnia* we used a device similar to that designed by Dawidowicz & Loose (1992)^33^ and Loose & Dawidowicz (1994)^34^, called a “plankton organ”, to track the diel vertical migration behavior of individual animals. It consisted of vertical glass tubes (1 m in length, 1 cm in diameter) set parallel to one another in a thermally stratified transparent water bath (20.0 °C at the top, 10.0 °C at the bottom). The setup was illuminated by halogen lamps shining overhead through a frosted glass diffuser, on a summer photoperiod (16 L:8D) setting.

In the **ox-bile** experiments, we tested four different media: the control medium, which was 0.45-μm filtered water from the Szczęśliwice reservoir; the fish medium (F), which was prepared by keeping a single crucian carp (7 cm total length) in 10 L of pre-filtered control water for 24 hours (equivalent to 0,1 fish L^−1^); the bile salts (BS) medium, which was a 0.001% solution of bile salts diluted in the pre-filtered control water; and Ox – ox bile medium, a 0.001% solution of ox-bile in the same water.

In the **fish-bile** experiments, we tested four different media: the control medium which was 0.45-μm filtered water from the Szczęśliwice reservoir; the fish medium (F), which was prepared by keeping a single crucian carp (7 cm total length) in 10 L of pre-filtered control water for 24 hours (equivalent to 0,1 fish L^−1^); and a fish bile medium, a 0.0001% v/v solution of bile extracted from the gallbladder freshly dissected from euthanized: a) the same crucian carp that was used to produce kairomone, b) common rudd *Scardinius erythrophthalmus* (7–8 cm) and diluted in the pre-filtered control water.

Sixty (in the ox-bile experiment) and forty five *D. magna* (in the fish-bile experiment) were simultaneously exposed to each of these media in 12 tubes (5 individuals per tube × 3 replicate tubes × 4 tested media and 5 individuals per tube × 3 replicate tubes × 3 tested media, respectively). *Daphnia* were introduced to the tubes at noon, 8 h before a simulated sunset. The next midday, 8 h after “sunrise”, the depth distribution of the animals in each tube was determined by visual counting. This procedure was repeated 3 times, each time with a new set of *Daphnia* exposed in freshly prepared media, giving a total of 45 depth-counts per medium treatment.

### Statistical analysis

Statistical analysis was performed on the pooled results of the experimental repetitions. All results are presented as boxplots bisected at the median value with minimal and maximal whiskers. We used a non-parametric Kruskall-Wallis ANOVA to check whether the medians differed significantly between treatments and checked for differences between pairs of treatments using Dunn’s multiple comparison post-hoc test. Letters on the figures indicate statistically significant differences between the groups. Results of the Kruskall-Wallis test (H and *p*) as well as the *p*-value of Dunn’s post hoc test are presented in the tables S2–S5 in Supporting information. All calculations were made using the GraphPad Prism 5.0 program.

### Chromatography

We performed chromatographic analyses of fish conditioned water, lyophilized ox bile and bile of a cyprinid fish in order to compare their bile acids/salts content.

### Enrichment and extraction of fish kairomones from fish conditioned water

Twenty fish (*Carassius auratus*) were kept in 10 L of water for 24 hours, with no food added. The kairomone to be used for chromatographic analysis was then extracted from the water by solid-phase extraction (SPE), following a procedure initially developed and used by von Elert & Loose (1996)^6^. Briefly, prior to adding the sample C18-SPE cartridges (500 mg, Analytichem Int.) were preconditioned with methanol and ultrapure water (10 ml each). The pH of the sample was adjusted to 7.0 with 2 M HCl. Methanol was added to achieve a 1% concentration. The resultant solution was then passed through the cartridge and the eluate was collected. The loaded cartridge was washed with 5 ml of ultrapure water prior to elution of the isolate with 10 ml of methanol (eluent). Both the eluate and the eluent were evaporated separately until dry using a rotatory evaporator to remove organic solvents, and then resuspended in methanol. Finally, the pooled eluate + eluent was evaporated until dry and resuspended in 1 ml of methanol.

### Obtaining fish bile for chromatographic analysis

Fish bile was obtained from one adult carp (*Cyprinus carpio*) which was purchased from a fish wholesaler. Euthanized carp was kept on ice and whole bile was aspirated directly from the gallbladder with a sterile syringe and needle. The aspirated bile (around 1 ml) was then lyophilized until dry to yield a greenish powder. This material was then dissolved in 10% methanol and used for chromatographic analyses.

All experimental protocols, methods and procedures used in the described experiments were performed in accordance with the regulations of the Polish Ethical Council for the care and use of laboratory animals, the European Community Directive for the ethical use of experimental animals, I Local Ethics Committee in Warsaw and Ordinance of minister of agriculture and rural development of December 14, 2016. In our experiment, we kept fish in the aquarium, and then fished them out, sacrificed in accordance with the guidelines approved by the I Ethics Committee in Warsaw to dissect the gallbladder.

### Chromatographic analyses

The chromatographic system was previously described by Biesaga *et al*. ^35^ for polyphenols analysis. The method we used, briefly described below, has been adapted for the compounds studied in our work. Chromatographic analysis was performed using a Shimadzu LC system consisting of LC20-AD binary pumps, a DGU-20A5 degasser, a CTO-20AC column oven and a SIL-20AC autosampler connected to a 3200 QTRAP mass spectrometer (Applied Biosystem/MDS SCIEX). An electrospray ionization(ESI) was operated in negative-ion mode. ESI conditions were as follows: capillary temperature 450 °C, curtain gas at 0.3 MPa, auxiliary gas at 0.3 MPa, negative ionization mode source voltage – 4.5 kV. Nitrogen was used as curtain and auxiliary gas. For each compound, the optimum conditions of Single Reaction Mode (SRM) were determined in infusion mode (see Supporting information Table S1). A standard solution of cholic acid from an ox bile dissolved in water was infused into the electrospray source with a 50 μm i.d. PEEK capillary using a Harward Apparatus pump at 10 μL/min. Continuous mass spectra were obtained by scanning m/z from 50 to 650.

Compounds were separated on a Luna (Phenomenex) C-18 (2) column (100 × 2.1 mm, 3 µm), with the precolumn set at 30 °C. We used 8 mM of formic acid (pH 2.8) as eluent A and acetonitrile as eluent B. The mobile phase was delivered at 0.2 mL/min in gradient mode: 0–3 min. 40% B, 3–20 min 70% B, 20–25 min 70% B, 26 min 40% B. Compounds were identified by comparing retention times and the m/z values obtained by mass spectra and SRM modes, with the mass spectra and SRM modes from standards (ox bile) tested under the same conditions. The obtained results were compared with data from Scherer *et al*. ^36^.

## Supplementary information

Supplementary Information.

## Data Availability

If the manuscript is accepted the data supporting the results will be archived in an appropriate public repository (Dryad) and the data DOI will be included at the end of the article.
