# Supplementary material for: Kairomone-like activity of bile and bile components: A step towards revealing the chemical nature of fish kairomone
Source: Sci Rep. 2020 Apr 27;10:7037. doi: 10.1038/s41598-020-63456-z (PMC7184620; doi:10.1038/s41598-020-63456-z)
Supplement: Supplementary file 1 — Supplementary Information. [file 41598_2020_63456_MOESM1_ESM.pdf]

Kairomone-like activity of bile and bile components: A step towards revealing the chemical nature of fish kairomone

Joanna Pijanowska, Magdalena Markowska, Anna Ruszczyńska, Ewa Bulska, Piotr Dawidowicz, Mirosław Ślusarczyk and Magdalena Biesaga

Table S1

LC/MS/MS characteristics of bile acids from ox bile; DP – declastering potential, CE – collision energy Q1 and Q3 - ion mass to charge ratio (m/z) chosen in the first and the third quadrupole (quadrupole mass filters), respectively (corrected MB)

Compound	Retention time, min	Q1, amu	Q3, amu	DP, V	CE, V
GCA	5,26	464,2	74,1	-100	-64
CA	9,15	407,1	345,1	-105	-40
CDCA/UDCA, DCA/ HDCA	12,4; 16,14	391,1	345	-90	-40
TCA	13,92	514,1	79,8	-100	-108
GCDCA/ GDCA/ CHDCA	10,44; 11,41	448,2	74,1	-100	-62
TDCA/TCDCA	24,64; 28,50	498,2	79,8	-100	-102

Table S2

Results of Kruskall-Wallis (H and p) as well as p of Dunn`s post hoc test of data presented in the Figure 1

	numer of individuals (n)	Kruskall-Wallis H; # of groups = 3	Kruskall-Wallis *p*	Dunn`s *p*
size at first reproduction	30-35	7,388	0,0249	Ox *vs* control <0,05
age at first reproduction	22-25	1,325	0,5155	ns
offspring number	30-34	7,787	0,0204	Ox *vs* BS <0,05
body length of neonates	309-389	184,6	<0,001	Ox *vs* control <0,001<br>BS *vs* control <0,001<br>Ox *vs* BS <0,001
reproductive effort	30-33	3,705	0,0988	ns

Table S3

Results of Kruskall-Wallis (H and p) as well as p of Dunn`s post hoc test of data presented in the Figure 2.

	numer of individuals (n)	Kruskall-Wallis H; # of groups = 5	Kruskall-Wallis *p*	Dunn`s *p*
size at first reproduction	6-8	20,04	0,005	AS+F *vs.* control <0,01<br>AS+Ox *vs.* control <0,01
offspring number	5-8	7,19	0,1259	ns
body length of neonates	39-83	106,6	<0,0001	AS *vs.* control <0,001<br>AS+F *vs.* control <0,01<br>AS+Ox *vs.* control <0,01<br>AS+BS *vs.* control <0,01<br>AS *vs.* AS+Ox <0,001<br>AS+F *vs.* AS+BS <0,01<br>AS+Ox *vs.* AS+BS <0,001
reproductive effort	5-8	6,029	0,1970	ns

Table S4

Results of Kruskall-Wallis (H and p) as well as p of Dunn`s post hoc test of data presented in the Figure 3.

	numer of individuals (n)	Kruskall-Wallis H; # of groups = 4	Kruskall-Wallis *p*	Dunn`s *p*
vertical distribution	41-44	28,80	<0,0001	F *vs* control <0,001<br>Ox *vs* control <0,01<br>BS *vs* control <0,01

Table S5

Results of Kruskall-Wallis (H and p) as well as p of Dunn`s post hoc test of data presented in the Figure 4.

	numer of individuals (n)	Kruskall-Wallis H; # of groups = 4	Kruskall-Wallis *p*	Dunn`s *p*
vertical distribution	15-44	43,44	<0,0001	F *vs* control <0,001<br>crucian carp *vs* control <0,001<br>common rudd *vs* control <0,01
